# Trans-Gastric Versus Trans-Duodenal Endoscopic Ultrasound-Guided Gallbladder Drainage: Which Is the Optimal Access Route?

**DOI:** 10.3390/biomedicines14071429

**Published:** 2026-06-24

**Authors:** Serena Stigliano, Claudia Marinaccio, Benedetto Neri, Nicolò Citterio, Marta Pettinelli, Dario Biasutto, Francesco Maria Di Matteo

**Affiliations:** 1 Therapeutic GI Endoscopy Unit, Fondazione Policlinico Universitario Campus Bio-Medico, 00128 Rome, Italybenedettoneri@gmail.com (B.N.); marta.pettinelli@unicampus.it (M.P.); d.biasutto@policlinicocampus.it (D.B.); 2 Gastroenterology and Endoscopy Unit, Campus Bio Medico University of Rome, 00128 Rome, Italy; 3 Università Campus Bio-Medico di Roma, Via Alvaro del Portillo, 21, 00128 Roma, Italy

**Keywords:** endoscopic ultrasound, EUS-guided gallbladder drainage, EUS-GBD, trans-gastric access, trans-duodenal access

## Abstract

**Background/Objectives**: Endoscopic ultrasound-guided gallbladder drainage (EUS-GBD) with Lumen-Apposing Metal Stent (LAMS) is an established option for high-surgical-risk patients, with high technical and clinical success. Indications include acute cholecystitis and palliation of jaundice in malignant distal biliary obstruction (MDBO). Both trans-gastric and trans-duodenal approaches are used, but the optimal route remains debated. The aim of the study was to compare trans-gastric and trans-duodenal access in terms of technical success, adverse events, readmissions, and reinterventions. **Methods**: We implemented a single-centre retrospective study of consecutive EUS-GBD procedures with LAMS at a tertiary endoscopy unit (January 2020–January 2026). Demographic, clinical, and procedural data were analyzed using appropriate statistical tests. **Results**: Seventy patients were included (51.4% male; mean age 77 ± 12 years). Indications were acute cholecystitis (64.3%) and MDBO (35.7%). Trans-gastric access was used in 48.5% of cases. A Hot-Axios LAMS was deployed in 77.2% of cases, mostly >10 mm. Technical success was achieved in 98.5% of cases. Naso-cystic drainage (NCD) was used through the LAMS in 47.1% of patients, while a double pig-tail plastic stent was used in 7.2% of patients. Adverse events were rare (1.4% misdeployment). LAMS obstruction occurred in 10% of patients, with reintervention required in 12.8% of patients. No differences were found between access routes in indication, technical success, LAMS type/size, or adjunctive drainage. However, trans-gastric access was associated with a higher reintervention rate (*p* = 0.01). **Conclusions**: EUS-GBD is a safe and effective procedure. While both approaches are comparable in most outcomes, the trans-gastric route may carry a higher risk of reintervention and should be avoided when alternative access is feasible.

## 1. Introduction

EUS-guided gallbladder drainage (EUS-GBD) has emerged as a preferred alternative to percutaneous and trans-papillary drainage for acute cholecystitis patients at high surgical risk, offering several proven advantages [1,2,3,4].

Additionally, EUS-GBD has been indicated as a rescue procedure in patients with unresectable malignant distal biliary obstruction (MDBO) when conventional endoscopic retrograde cholangiopancreatography (ERCP) and EUS-guided biliary drainage fail or are not feasible, offering effective palliation and biliary decompression [1,5,6,7]. A recent meta-analysis including 13 studies using LAMS in high-risk surgical patients with acute cholecystitis or biliary obstruction reported technical and clinical success rates of EUS-GBD of 94% and 93% respectively [8].

Despite being considered a safe procedure in centres with expertise, EUS-GBD shows a wide range of adverse event rates (8–26%) with bleeding, stent migration, stent occlusion and capnoperitoneum being the most frequent [9,10]. Predictive factors for EUS-GBD failure or complications have still not been identified, although severe inflammation, ascites and absence of gallbladder apposition have been suggested [11]. Moreover, data on the long-term risk of LAMS obstruction, the need for reintervention, rates and types of adverse events, and the burden of rehospitalization are still conflicting [12,13].

Both trans-gastric and trans-duodenal drainage have been reported. On one hand, the trans-duodenal approach could be preferred considering that the position of the stent may be less affected by peristalsis, potentially lowering the risk of food impaction. On the other hand, the trans-gastric approach could be preferred for patients who may be considered for cholecystectomy in future.

Few studies comparing trans-gastric with trans-duodenal EUS-GBD have been reported, and which is the best approach is still controversial [14]. Therefore, the aim of the present study was to compare the trans-gastric and trans-duodenal approach of EUS-GBD in terms of technical and clinical success, the rate of adverse events, unplanned readmissions and reinterventions.

## 2. Materials and Methods

In this single-centre retrospective study, all consecutive cases of EUS-GBD with LAMS performed at the Therapeutic Endoscopy Unit of Fondazione Policlinico Universitario Campus Bio-Medico of Rome were considered. Both patients with acute cholecystitis or with malignant biliary obstruction were included. Patients’ demographic and clinical data, LAMS type and size and procedural characteristics were recorded. The rate of early and late adverse events was recorded and classified according to the AGREE classification [15].

Technical success was defined as correct deployment of LAMS. Clinical success, for patients with acute cholecystitis was defined as resolution of symptoms and improvement of inflammatory markers within 72 h from the procedure. For those with MDBO, the clinical success was defined as either a ≥50% reduction in serum total bilirubin or a decrease to below 3 mg/dL within two weeks.

All planned and unplanned secondary endoscopic procedures performed after EUS-GBD aimed to address clinical failure, recurrent symptoms, or device complications were considered as reinterventions.

### 2.1. Procedure

All EUS-GBD procedures were performed with fluoroscopy under deep sedation. A therapeutic linear echoendoscope (EG-580UT, Fujifilm Corp., Tokyo, Japan) and a specific ultrasound observation system (ARIETTA 850; FUJIFILM Healthcare, Tokyo, Japan) were used for all cases. LAMS included the AXIOS Stent and Electrocautery Enhanced Delivery System; Boston Scientific, Marlborough, MA, USA) and the Hot-Spaxus (Taewoong Medical, Gimpo-si, South Korea).

### 2.2. Ethical Approval

This study was approved by the Territorial Ethic Committee of Lazio Area 2 (81.23OSS). Patient consent was waived as data were retrospectively collected, the investigation did not add risk for participants, and all data were de-identified.

### 2.3. Statistical Analysis

Continuous variables were expressed as mean ± standard deviation or median and interquartile range (IQR), depending on data distribution. Categorical variables were presented as absolute numbers and percentages. Categorical differences were assessed using the Chi-square or Fisher’s exact test, with Yates’ correction applied as appropriate. Continuous data were compared by using the unpaired Student’s t-test for parametric variables and the Mann–Whitney U test for non-parametric variables. Statistical significance was set at *p* < 0.05. All analyses were performed using IBM SPSS Statistics, version 26.0.

## 3. Results

### 3.1. Study Population

**Figure 1:**
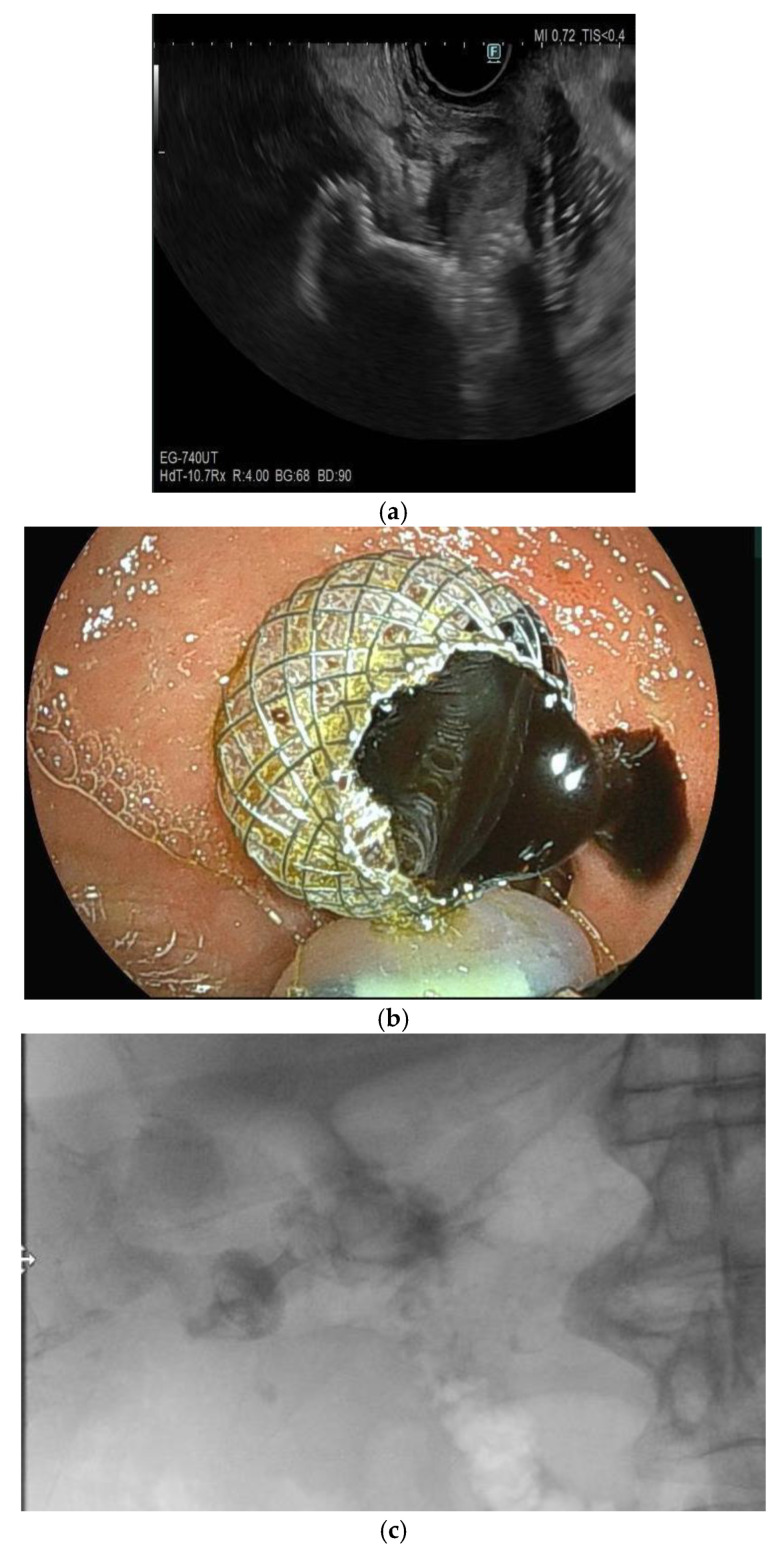
(**a**) Endoscopic ultrasound image of gallbladder drainage with LAMS, with distal flange opened correctly inside the gallbladder and proximal flange in the GI lumen. (**b**) Endoscopic image of proximal flange open in the GI lumen with bile flow. (**c**) Fluoroscopy image of LAMS deployment in correct position.

From January 2020 to January 2026, seventy patients were enrolled (51.4% male; 77 ± 12 years old). Indications for EUS-GBD included acute cholecystitis in 45 (64.3%) patients and MDBO in 25 (35.7%) patients. Overall, in 34 (48.5%) patients trans-gastric access was performed and in 36 (51.2%) patients trans-duodenal access was performed. In 54 (77.2%) patients a Hot-Axios LAMS was used and in 53 (75.7%) patients a calibre ≥ 10 mm was used (Table 1). Intrachannel release was performed in all cases. Overall technical success was observed in 69 (98.5%) procedures (Figure 1). In the subgroup of patients with acute cholecystitis, clinical success was observed in 39/45 (86.7%) and in those with MDBO in 16/25 (64%). In only one case was the LAMS dilated. Naso-cystic drainage (NCD) was used through the LAMS with 33 (47.1%) patients, while a double pig-tail plastic stent was used with five (7.2%) patients, all measuring 7Fr 3 cm.

**Figure 2:**
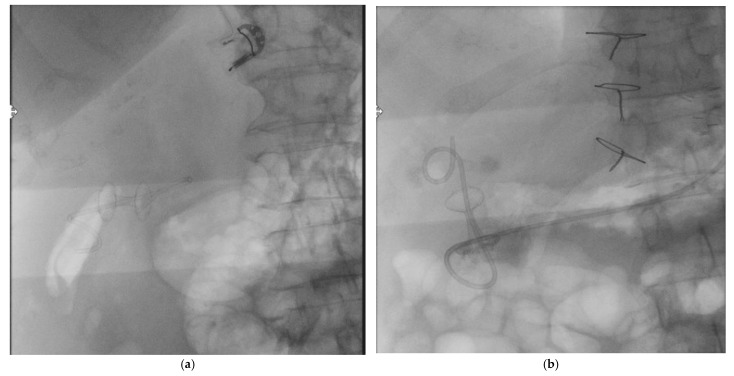
(**a**) Fluoroscopy image of endoscopic ultrasound-guided gallbladder drainage with Lumen-Apposing Metal Stent placement with a coaxial double pig-tail stent passing through. (**b**) Fluoroscopy image of endoscopic ultrasound-guided gallbladder drainage with Lumen-Apposing Metal Stent placement with naso-cystic drainage passing through.

The only adverse event observed occurred in one patient with distal flange LAMS misdeployment. The patient was successfully managed endoscopically by removing the LAMS, then closing the duodenal access with an over-the-scope clip and, as a subsequent CT scan did not report bile leak or peri-cholecystic fluid, with broad-spectrum antibiotics coverage. LAMS obstruction occurred in seven (10%) patients, while reintervention was required in nine (12.8%) patients. The causes of reintervention included LAMS obstruction (n = 7), pig-tail stent replacement (n = 1) and gallstone lithotripsy (n = 1) (Figure 2). In three (4.5%) patients, LAMS migration was observed with a mean time of 72 ± 93 days. Overall, the mean duration of hospital stay after EUS-GBD was 9 ± 7 days (Table 1). Overall, the median follow-up of the patients was 56 days with an interquartile range of 165 days.

### 3.2. Comparison Between Patients Undergoing EUS-GBD with Trans-Gastric and Trans-Duodenal Access

There was no statistical difference in terms of drainage indication (acute cholecystitis: 64.7% vs. 63.8%, *p* = 1), technical success rate (100% vs. 97.2%, *p* = 1), overall clinical success rate (82.4% vs. 79% *p* = 1), LAMS type (AXIOS: 79.4% vs. 75%, *p* = 0.78) and size (≥10 mm: 79.4% vs. 72.2%, *p* = 0.58) between the two different types of EUS-GBD access. In addition, comparable rates of NCD (55.8% vs. 38.8% *p* = 0.23) and double pig-tail plastic stent placement (8.8% vs. 5.5% *p* = 0.66) were observed.

The mean duration of hospitalization after the procedure also did not differ in patients receiving EUS-GBD with trans-gastric access compared with those with trans-duodenal access (10 ± 9 days vs. 8 ± 6 days, *p* = 0.24).

Trans-gastric access was not associated with a higher rate of LAMS misdeployment (0% vs. 2.7% *p* = 1), obstruction (17.6% vs. 2.7% *p* = 0.11) or migration rate (2.9% vs. 8.8% *p* = 0.61). However, an overall higher need for reintervention was observed in patients undergoing EUS-GBD with trans-gastric access (23.5% vs. 2.7% *p* = 0.02) (Table 2). Logistic regression analysis results confirmed a higher risk of reintervention when EUS-GBD was performed with trans-gastric access (OR 10 [95% CI 1.16–85], *p* = 0.03).

## 4. Discussion

Endoscopic ultrasound-guided gallbladder drainage with LAMS is a safe procedure with a high technical success rate. Several studies and meta-analyses have demonstrated the superiority of this technique over other GB drainage techniques (PT and ET-GBD). There are also some unresolved questions about the standardization of the technique, especially in terms of size of the LAMS and position (trans-gastric or trans-duodenal).

This study therefore aimed to compare the two different access routes with respect to technical success, adverse event rates, unplanned readmissions, and reinterventions.

Both trans-duodenal and trans-gastric EUS-GBD offer theoretical advantages. The duodenum is relatively fixed and lies closer to the gallbladder than the stomach, which may reduce the risk of stent migration. Additionally, trans-duodenal GBD may be associated with a lower likelihood of food reflux. On the other hand, trans-gastric access is technically easier due to the larger diameter of the gallbladder body, which also facilitates deployment of the inner flange of the LAMS. Furthermore, if the procedure fails, rescue surgery is generally more straightforward after attempted trans-gastric drainage than after a trans-duodenal approach [16,17,18,19,20,21]. Trans-duodenal access is usually preferred over trans-gastric EUS-GBD, as antral LAMS placement has been associated with greater symptom recurrence owing to food impaction and a higher risk of a buried LAMS [22,23,24].

In patients undergoing EUS-GBD as a bridge to cholecystectomy, the subsequent elective surgery can be technically challenging. Some surgeons find closure of a trans-duodenal defect more difficult than that of trans-gastric access. Conversely, the fibrous adhesion that may arise between the stomach and the gallbladder after trans-gastric drainage may represent a major obstacle during later cholecystectomy [25]. Current evidence suggests that both trans-gastric and trans-duodenal EUS-GBD are characterized by comparable safety and efficacy.

In our study population there was no difference in terms of technical success and adverse event rates between the two different access types for EUS-GBD. Furthermore, the mean time of hospitalization after the procedure was also comparable.

However, the trans-gastric approach was associated with a higher need for reintervention (23.5% vs. 2.7%), suggesting a preference for the trans-duodenal approach. Nevertheless, this is not always feasible, and the choice of drainage route should be tailored to individual anatomical and technical factors.

Trans-gastric drainage is generally favoured in patients with duodenal malignant involvement or in those with an indwelling duodenal self-expandible metal stent. In other cases, the preferred approach should be selected according to the point of closest gallbladder apposition to the gastrointestinal lumen, the absence of intervening vessels at the puncture site, and the ability to maintain a stable echoendoscope position [26].

In our study population nearly half of the patients underwent NCD positioning at the time of EUS-GBD, as per local routinary practice. The rationale is to favour the drainage of the infected bile, potentially speeding up the recovery process. Currently, there are no robust data on the role of NCD placement. In the present study no differences were observed between patients with versus without NCD placement in terms of hospitalization duration (11 ± 9 vs. 7 ± 6 days, *p* = 0.12), rate of LAMS obstruction (28.6% vs. 50% *p* = 0.45) and reintervention (54.5% vs. 46.1% *p* = 0.74), thus the clinical usefulness of this procedure cannot be deduced.

In the present study, the clinical success rate of EUS-GBD for acute cholecystitis was high (86.7) and comparable to rates currently reported in the literature. Indeed, in the most recent metanalysis a 94.3% clinical success rate was suggested [27]. This rate is slightly higher than that observed in our population; however the definition adopted was different since we assessed symptoms and biochemical improvement 72 h after the procedure while the aforementioned paper made the assessment after 4 weeks. In contrast, a lower clinical success rate (64%) has been observed in a subgroup of patients undergoing EUS-GBD for MDBO when compared to most recent data. However, in the largest and most recent metanalysis a broad range of clinical success rates has been suggested (77.8–100%), with significant variability in the definition used [13]. The higher mean age of treated patients included in our cohort, potentially implying more advanced disease, and the small sample size may both account for this finding.

A key limitation of this study is its retrospective design, which may imply that the EUS-GBD approach and the selection of both the size and type of LAMS were left to the endoscopist’s discretion. However, this real-world scenario reflects routine clinical practice. Among the main strengths of this study is the fact that the enrolled cases were homogeneous, with comparable patient characteristics (age, sex, and indication for gallbladder drainage) between the two different approaches, thereby reducing potential biases.

In conclusion, our study confirms that EUS-GBD shows very low rates of early and late adverse events, independently of the access method used. However, since EUS-GBD with trans-gastric access seems to be associated with a higher rate of reintervention, when possible, another route should be used.

## Figures and Tables

**Table 1:** Study population demographic, clinical and procedural characteristics.

	EUS-GBD n = 70
**Male sex, n (%)**	36/70 (51.4%)
**Age, mean (±SD)**	77 ± 12
**Acute cholecystitis, n (%)**	45/70 (64.3%)
**Trans-gastric access, n (%)**	34/70 (48.5%)
**Hot-Axios LAMS, n (%)**	54/70 (77.2%)
**LAMS ≥ 10 mm**	53/70 (75.7%)
8 × 8 mm	14/70 (20%)
10 × 10 mm	46/70 (65.7%)
16 × 20 mm	1/70 (1.4%)
20 × 10 mm	9/70 (12.8%)
**NCD, n (%)**	33/70 (47.1%)
**Double pig-tail stent, n (%)**	5/70 (7.2%)
**Technical success, n (%)**	69/70 (98.5%)
**LAMS misdeployment, n (%)**	1/70 (1.4%)
**LAMS obstruction, n (%)**	7/70 (10%)
**Rate of reintervention, n (%)**	9/70 (12.8%)
**LAMS migration, n (%)**	3/70 (4.5%)
**Hospitalization duration, mean (±SD)**	9 ± 7 days

Abbreviations: LAMS: Lumen-Apposing Metal Stent; NCD: naso-cystic drainage, SD: standard deviation.

**Table 2:** Comparisons of demographic, clinical and procedural characteristics of patients undergoing different endoscopic ultrasound-guided gallbladder drainage approaches.

	EUS-GBD with Trans-Gastric Access	EUS-GBD with Trans-Duodenal Access	*p*
**Male sex, n (%)**	16/34 (47%)	20/36 (55.5%)	0.63
**Acute cholecystitis, n (%)**	22/34 (64.7%)	23/36 (63.8%)	1
**Hot Axios LAMS, n (%)**	27/34 (79.4%)	27/36 (75%)	0.78
**Calibre ≥ 10 mm, n (%)**	27/34 (79.4%)	26/36 (72.2%)	0.58
**Dilatation, n (%)**	1/34 (2.9%)	0/35 (0%)	0.49
**Technical success, n (%)**	34/34 (100%)	35/36 (97.2%)	1
**Clinical success n (%)**	28/34 (82.4%)	27/36 (75%)	0.9
**NCD, n (%)**	19/34 (55.8%)	14/36 (38.8%)	0.23
**Double Pig-tail stent, n (%)**	3/34 (8.8%)	2/36 (5.5%)	0.66
**LAMS obstruction**	6/34 (17.6%)	1/36 (2.7%)	0.11
**Reintervention rate**	8/34 (23.5%)	1/36 (2.7%)	**0.02**
**Age, mean (±SD)**	75 ± 12	79 ± 10	0.12
**Length of hospitalization, mean (±SD)**	10 ± 9	8 ± 6	0.24
**Migration rate, n (%)**	1/34 (2.9%)	3/34 (8.8%)	0.61
**Misdeployment rate, n (%)**	0/34 (0%)	1/36 (2.7%)	1

Abbreviations: EUS-GBD: endoscopic ultrasound-guided gallbladder drainage access; LAMS: Lumen-Apposing Metal Stent; NCD: naso-cystic drainage, SD: standard deviation.

## Data Availability

The raw data supporting the conclusions of this article will be made available by the authors on request.

## References

[1] van der Merwe S.W., van Wanrooij R.L.J., Bronswijk M., Everett S., Lakhtakia S., Rimbas M., Hucl T., Kunda R., Badaoui A., Law R. (2022). Therapeutic endoscopic ultrasound: European Society of Gastrointestinal Endoscopy (ESGE) Guideline. Endoscopy.

[2] McCarty T.R., Hathorn K.E., Bazarbashi A.N., Jajoo K., Ryou M., Thompson C.C. (2021). Endoscopic gallbladder drainage for symptomatic gallbladder disease: A cumulative systematic review meta-analysis. Surg. Endosc..

[3] Teoh A.Y.B., Kitano M., Itoi T., Pérez-Miranda M., Ogura T., Chan S.M., Serna-Higuera C., Omoto S., Torres-Yuste R., Tsuichiya T. (2020). Endosonography-guided gallbladder drainage versus percutaneous cholecystostomy in very high-risk surgical patients with acute cholecystitis: An international randomised multicentre controlled superiority trial (DRAC 1). Gut.

[4] Boregowda U., Chen M., Saligram S. (2023). Endoscopic Ultrasound-Guided Gallbladder Drainage versus Percutaneous Gallbladder Drainage for Acute Cholecystitis: A Systematic Review and Meta-Analysis. Diagnostics.

[5] Mangiavillano B., Moon J.H., Facciorusso A., Vargas-Madrigal J., Di Matteo F., Rizzatti G., De Luca L., Forti E., Mutignani M., Al-Lehibi A. (2024). Endoscopic ultrasound-guided gallbladder drainage as a first approach for jaundice palliation in unresectable malignant distal biliary obstruction: Prospective study. Dig. Endosc..

[6] Imai H., Kitano M., Omoto S., Kadosaka K., Kamata K., Miyata T., Yamao K., Sakamoto H., Harwani Y., Kudo M. (2016). EUS-guided gallbladder drainage for rescue treatment of malignant distal biliary obstruction after unsuccessful ERCP. Gastrointest. Endosc..

[7] Chang J., Dong E., Kwok K. (2019). Endoscopic ultrasound-guided transmural gallbladder drainage in malignant obstruction using a novel lumen-apposing stent: A case series (with video). Endosc. Int. Open.

[8] Kalva N.R., Vanar V., Forcione D., Bechtold M.L., Puli S.R. (2018). Efficacy and Safety of Lumen Apposing Self-Expandable Metal Stents for EUS Guided Cholecystostomy: A Meta-Analysis and Systematic Review. Can. J. Gastroenterol. Hepatol..

[9] Saumoy M., Novikov A., Kahaleh M. (2018). Long-term outcomes after EUS-guided gallbladder drainage. Endosc. Ultrasound.

[10] Martínez-Moreno B., López-Roldán G., Escuer J., Gornals J.B., Loras C., Gordo A., Vila J., Bazaga S., Durá M., Sanchiz V. (2025). Outcomes of a multicenter registry on EUS-guided gallbladder drainage as a rescue technique for malignant distal biliary obstruction after failed endoscopic retrograde cholangiopancreatography. Endosc. Ultrasound.

[11] Ishiwatari H., Sakamoto H., Doi T., Yamamura M. (2026). Prevention of Adverse Events in Endoscopic Ultrasound-Guided Biliary Drainage. DEN Open.

[12] Irani S.S., Sharma N.R., Storm A.C., Shah R.J., Chahal P., Willingham F.F., Swanstrom L., Baron T.H., Shlomovitz E., Kozarek R.A. (2023). Endoscopic Ultrasound-guided Transluminal Gallbladder Drainage in Patients With Acute Cholecystitis. Ann. Surg..

[13] Rizzo G.E.M., Crinò S.F., Vanella G., Facciorusso A., Fusaroli P., Catena F., Trieu J.A., Baron T.H., Anderloni A., Fabbri C. (2025). EUS-guided gallbladder drainage as a rescue in distal malignant biliary obstruction: A systematic review with meta-analysis. Endosc. Ultrasound.

[14] Sobani Z.A., Ling C., Rustagi T. (2021). Endoscopic Ultrasound-Guided Gallbladder Drainage. Dig. Dis. Sci..

[15] Nass K.J., Zwager L.W., van der Vlugt M., Dekker E., Bossuyt P.M., Ravindran S., Thomas-Gibson S., Fockens P. (2022). Novel classification for adverse events in GI endoscopy: The AGREE classification. Gastrointest. Endosc..

[16] Boregowda U., Umapathy C., Nanjappa A., Wong H., Desai M., Roytman M., Theethira T., Saligram S. (2018). Endoscopic ultrasound guided gallbladder drainage—Is it ready for prime time?. World J. Gastrointest. Pharmacol. Ther..

[17] Posner H., Widmer J. (2020). EUS guided gallbladder drainage. Transl. Gastroenterol. Hepatol..

[18] Chan J.H.Y., Teoh A.Y.B. (2018). Current Status of Endoscopic Gallbladder Drainage. Clin. Endosc..

[19] Itoi T., Itokawa F., Kurihara T. (2011). Endoscopic ultrasonography-guided gallbladder drainage: Actual technical presentations and review of the literature (with videos). J. Hepato-Biliary-Pancreat. Sci..

[20] Widmer J., Singhal S., Gaidhane M., Kahaleh M. (2014). Endoscopic ultrasound-guided endoluminal drainage of the gallbladder. Dig. Endosc..

[21] Crino S.F., Rimbaș M., Gabbrielli A., Larghi A. (2019). Endoscopic Ultrasound Guided Gallbladder Interventions: A Review of the Current Literature. J. Gastrointest. Liver Dis..

[22] Chan S.M., Teoh A.Y.B., Yip H.C., Wong V.W.Y., Chiu P.W.Y., Ng E.K.W. (2017). Feasibility of per-oral cholecystoscopy and advanced gallbladder interventions after EUS-guided gallbladder stenting (with video). Gastrointest. Endosc..

[23] Cho S.H., Oh D., Song T.J., Park D.H., Seo D.-W., Lee S.K., Kim M.-H., Lee Y.N., Moon J.H., Lee S.S. (2020). Comparison of the effectiveness and safety of lumen-apposing metal stents and anti-migrating tubular self-expandable metal stents for EUS-guided gallbladder drainage in high surgical risk patients with acute cholecystitis. Gastrointest. Endosc..

[24] Dollhopf M., Larghi A., Will U., Rimbaş M., Anderloni A., Sanchez-Yague A., Teoh A.Y.B., Kunda R. (2017). EUS-guided gallbladder drainage in patients with acute cholecystitis and high surgical risk using an electrocautery-enhanced lumen-apposing metal stent device. Gastrointest. Endosc..

[25] Perez-Miranda M. (2018). Technical considerations in EUS-guided gallbladder drainage. Endosc. Ultrasound.

[26] Rana S.S. (2021). Endoscopic ultrasound-guided gallbladder drainage: A technical review. Ann. Gastroenterol..

[27] Canakis A., Tugarinov N., Deliwala S., Twery B., Miro-Gonzalez Á., Beran A., Gorman E.F., Hathorn K., Gilman A.J., Chawla S. (2026). Clinical outcomes of Endoscopic ultrasound-guided gallbladder drainage in patients with acute cholecystitis with ≥1 year of follow-up: A systematic review and meta-analysis. Gastrointest. Endosc..

